# Monitoring of Dynamic Microbiological Processes Using Real-Time Flow Cytometry

**DOI:** 10.1371/journal.pone.0080117

**Published:** 2013-11-14

**Authors:** Markus Arnoldini, Tobias Heck, Alfonso Blanco-Fernández, Frederik Hammes

**Affiliations:** 1 Department of Environmental Microbiology, Eawag - Swiss Federal Institute for Aquatic Science and Technology, Dübendorf, Switzerland; 2 Department of Environmental Systems Science, ETH Zürich, Zürich, Switzerland; 3 Laboratory for Biomaterials, Empa -Swiss Federal Laboratories for Materials Science and Technology, St. Gallen, Switzerland; 4 Flow Cytometry Core Facilities, UCD-Conway Institute of Biomolecular & Biomedical Research, University College Dublin, Dublin, Ireland; Texas Tech University Health Sciences Center, United States of America

## Abstract

We describe a straightforward approach to continuously monitor a variety of highly dynamic microbiological processes in millisecond resolution with flow cytometry, using standard bench-top instrumentation. Four main experimental examples are provided, namely: (1) green fluorescent protein expression by antibiotic-stressed *Escherichia coli*, (2) fluorescent labeling of heat-induced membrane damage in an autochthonous freshwater bacterial community, (3) the initial growth response of late stationary *E. coli* cells inoculated into fresh growth media, and (4) oxidative disinfection of a mixed culture of auto-fluorescent microorganisms. These examples demonstrate the broad applicability of the method to diverse biological experiments, showing that it allows the collection of detailed, time-resolved information on complex processes.

## Introduction

Flow cytometry (FCM) is a powerful and flexible method to characterize hydrodynamically focussed particles based on light scattering and fluorescence emission, thereby combining large sample sizes with considerable speed of data acquisition and ample information on single-particle level. FCM is used increasingly for a wide range of biotic and abiotic applications in the fields of medical research, biotechnology and environmental microbiology [1-3]. A typical FCM analysis involves single-point measurements of a selected sample volume, where the cumulative data of a measurement describes the state of the sample during the time of this measurement. However, during the course of any FCM measurement the sample flows continuously through the light source, which means that all collected data are automatically resolved in time. This feature allows assessment of changes occurring in the sample with sub-second resolution. Adding a temporal dimension to the collected information makes flow cytometry extremely powerful for monitoring dynamic changes in suspended cells without losing the single-particle resolution. Studying such processes with conventional techniques is only possible either by compromising on sample size, e.g. in time-lapse microscopy, or by losing the single-cell resolution, as it is the case in biochemical analyses, where large numbers of cells are pooled to yield a population average. Such a “real time” or “kinetic” FCM approach (from here on referred to as real-time flow cytometry, RT-FCM) was first applied by Martin and Swartzendruber [4], and has since been used for studying amongst other things the biochemical properties of mammalian cells [5-9] and protists [10], and interactions between abiotic molecules [1]. Nonetheless, given its immense potential, RT-FCM is surprisingly under-utilised in research applications. Recent technical advances in standard flow cytometry instrumentation have made it possible to perform such experiments using small and comparatively cheap devices, in standard research laboratories. 

The purpose of the present study is to illustrate the broad possibilities that this RT-FCM approach offers to collect information on a variety of parameters, potentially providing a tool to answer a number of different research questions on diverse study objects. For this reason we opted for a series of demonstrative experiments that highlight the application of these methods in everyday research, with particular emphasis on microbiological applications.

## Results

### Induction of the *E. coli* SOS response

**Figure 1:**
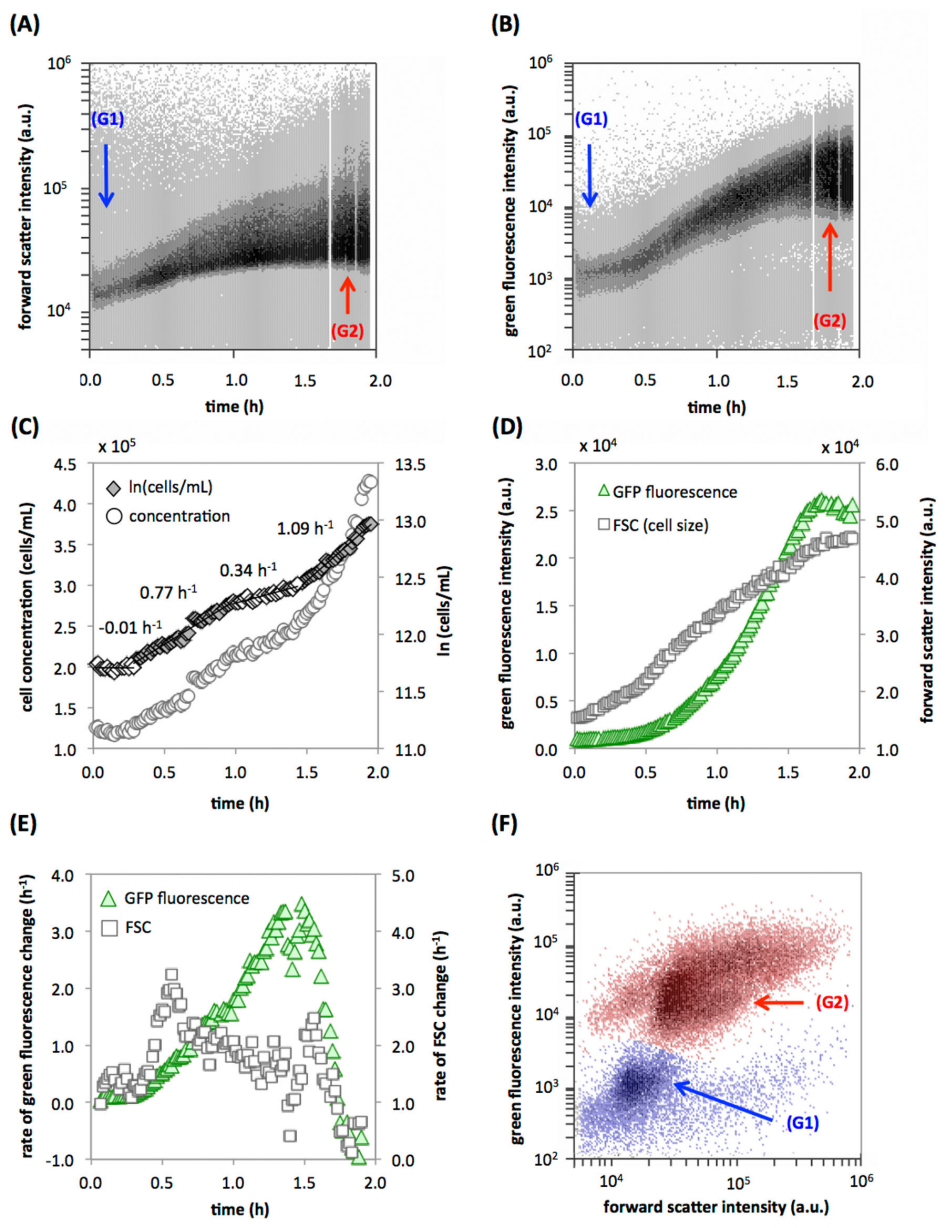
Induction of RecA transcription and GFP expression in *E. coli* cells exposed to 4 µg/ml ciprofloxacin. (**A**) Raw data of forward scatter (FSC) intensity over time, with marked regions (G1) and (G2) showing the data used for panels B and F. (**B**) Raw data of bacterial GFP fluorescence intensity (ex. 488 nm/em. 530 nm) over time. (**C**) Processed data (1-minute intervals) of cell concentrations during the experiment, showing four stages with varying growth rates. (**D**) Quantified changes in median cell size (forward scatter) and GFP fluorescence intensity. (**E**) Calculated rates of change in cell size and fluorescence intensity. (**F**) FCM overlay density plot of the culture at the start (blue data; (G1)) and end (red data; (G2)) of the experiment (from panel A). Gating procedures and calculations are detailed in the Materials and Methods section.

We monitored induction of the SOS response in *E. coli*, using expression of green fluorescent protein (GFP) controlled by the promoter of *recA* as reporter. RecA is a major regulator of the *E. coli* SOS response, and its expression is induced upon DNA damage [11]. To induce the response, we added a sub-lethal dose of the gyrase inhibitor ciprofloxacin to the culture. Ciprofloxacin causes DNA damage and consequently activation of the *recA* promoter [12,13]. Figure 1A and 1B show the raw data of forward scatter (FSC) as indicator of cell size, and green fluorescence intensity as indicator of GFP expression, respectively, during two hours, equaling a total of approximately one million measured cells. From such data sets, a range of biologically relevant and quantifiable information for multiple variables can be extracted. As first basic information, Figure 1C shows the changes in the cell concentration at 1-minute resolution. After a short lag phase of about 15 minutes (growth rate (μ) = -0.01 h^-1^), an increase in the cell concentration was recorded throughout the experiment at a mean growth rate of 0.66 h^-1^ (Figure 1C), showing that cell division occurred in the presence of the antibiotic. Berney and coworkers [14] conducted detailed batch growth experiments for *E. coli* under similar conditions but without antibiotics, showing no apparent lag phase and initial growth rates between 1.5 - 2 h^-1^, thus clearly highlighting the effect of the antibiotics in the present experiment. Moreover, the high-resolution data elucidates three apparent stages with varying growth rates of 0.77 h^-1^, 0.34 h^-1^ and 1.09 h^-1^, respectively, although the reason for the varying growth rates is not clear. Induction of the *recA* gene as measured by average intracellular GFP fluorescence intensity was clearly quantifiable, with a clear increase between 0.5 - 1.75 hours, reaching a plateau of maximal fluorescence intensity shortly after that (Figure 1D). Relative cell size (measured as FSC) increased throughout the experiment due to cell elongation that is known to occur upon DNA damage [15], with a concomitant increase of *recA* induction in larger cells. Figure 1E shows the corresponding rates of change for GFP fluorescence and FSC intensity (calculated from the data in Figure 1D). These results suggest the highest rate of cell elongation shortly after addition of the antibiotic (between 0.4 - 0.8 h), decreasing thereafter as the cells start to respond to the damage. The rate of *recA* promoter activity (measured as GFP fluorescence), increased continuously and peaked at approximately 1.3 - 1.7 h. The delayed increase in GFP fluorescence, as compared to the increase in FSC, can be rationalized by the folding time of the GFP variant used in this experiment, which has been reported to be approximately 10 minutes [16]. Notably, the continuous data obtained by the RT-FCM approach allowed high resolution assessment of the kinetics of this process, revealing the specific changes in growth rates (Figure 1C) and changes in the rates of GFP expression and cell elongation (Figure 1E) that would likely be missed with a manual sampling approach. Moreover, the RT-FCM approach allows complete access to the experimental data collected at any specific time. Figure 1F shows an overlay dot-plot of events extracted from the acquired data set at approximately 5 minutes and 110 minutes, respectively, revealing a shift from a rather homogenous cell population at the start of the culture, to visible subpopulations after long exposure to ciprofloxacin. This experiment demonstrates the power of combining single-cell resolution and big sample sizes. Single-cell microbiology has gained significant attention in recent years, and phenotypic heterogeneity in clonal populations has been shown to be a common phenomenon, especially upon induction of genes associated with stress responses [17-19]. Here we show that the RT-FCM approach is perfectly suited for studying the time dependent dynamics of differentiation on a single cell level into heterogeneous subpopulations in a variety of experimental setups.

### Detection of temperature-induced cell damage using fluorescent dyes

**Figure 2:**
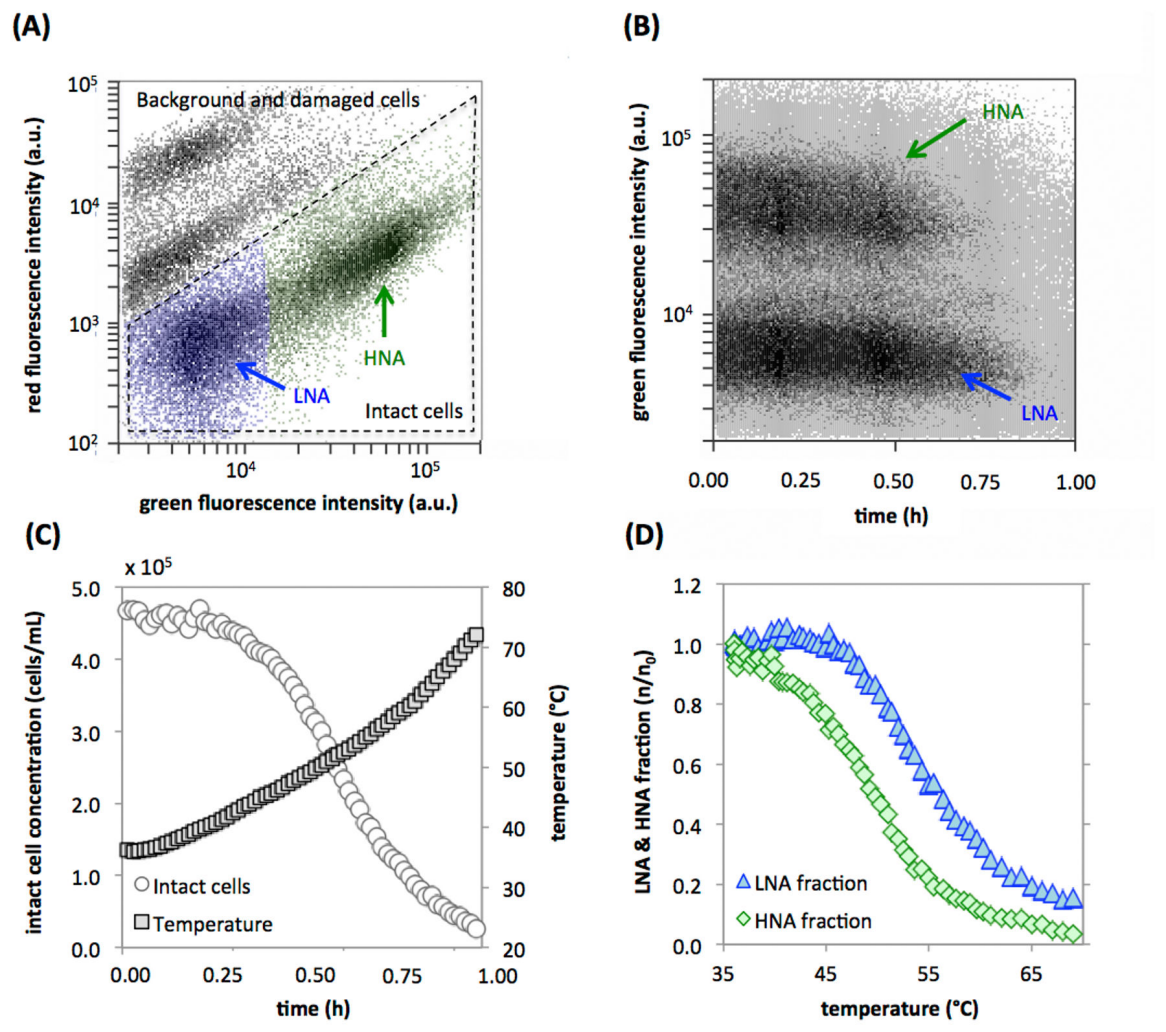
Temperature induced cell damage in a river water sample heated from 35 - 70 °C. (**A**) Density plot of the raw water stained with SYBR Green I and propidium iodide, showing the regions where “intact” and “damaged” cells are located, as well as the clusters of low (LNA; blue) and high (HNA; green) nucleic acid content bacteria. (**B**) Raw RT-FCM data of green fluorescence (ex. 488 nm/em. 530 nm) intensity over time for the intact cells (from panel A). (**C**) Processed data (1-minute intervals) showing the decrease in intact cell concentrations during the experiment directly linked to the increase in temperature. (**D**) Relative changes of the concentrations of the LNA (blue triangles) and HNA (green diamonds) bacterial fractions against temperature, showing a higher sensitivity to temperature in the HNA population.

In a second example, the assessment of bacterial membrane damage was illustrated by exposing an indigenous microbial community from a river water sample to continuously increasing temperatures. The purpose of this example is to demonstrate the applicability of the RT-FCM methodology in combination with fluorescent dyes to study the behavior of complex microbial communities in an environmental sample subjected to heat stress. A river water sample was heated from 35 °C to 70 °C in the presence of SYBR Green I and propidium iodide. SYBR green permeates bacterial cell membranes irrespective of their viability state and thus leads to staining (green fluorescence) of the entire microbial community. Propidium iodide is meant to permeate damaged membranes and is commonly used as an indicator for cellular damage (red fluorescence). When cells are damaged, a shift occurs from primarily green fluorescent cells to primarily red fluorescent cells [20,21]. As with any so-called viability dye, care should be taken since the propidium iodide has been reported to stain viable cells of certain bacterial strains during specific growth periods [22], and membrane permeability is just one aspect of bacterial viability and cell damage [23]. Figure 2A shows a density plot of the sample at time 0, with the distinctive, so-called low (LNA) and high (HNA) nucleic acid content cells commonly observed in natural freshwater environments [24]. In addition, Figure 2A shows the gating strategy used to separate intact cells from damaged cells and background. Figure 2B shows the density of green fluorescent events in the “intact cell” fraction from Figure 2A for the entire experiment. Note that the decrease in the density of green fluorescent particles does not indicate a loss of green fluorescence, but rather a shift of cells out of the gate containing intact cells when their red fluorescence increases as their membranes become increasingly compromised. Figure 2C and 2D show the detailed data (1-minute resolution) on cell integrity as a function of time and temperature respectively. It is evident that the number of intact cells remain essentially constant as long as the temperature was below 42 - 44 °C, after which cells are gradually damaged with increasing temperature and time (Figure 2C). Moreover, Figure 2D shows that the kinetics of cell damage of the LNA and HNA fractions are different, with the HNA fraction displaying membrane damage significantly faster than the LNA fraction. Such a dissimilar damage-response of the LNA and HNA bacterial fractions has been described previously for some oxidants [21] but not for heat treatment, though the correct explanation for this phenomenon remains to be elucidated.

### Initial bacterial response to fresh nutrients during lag phase

**Figure 3:**
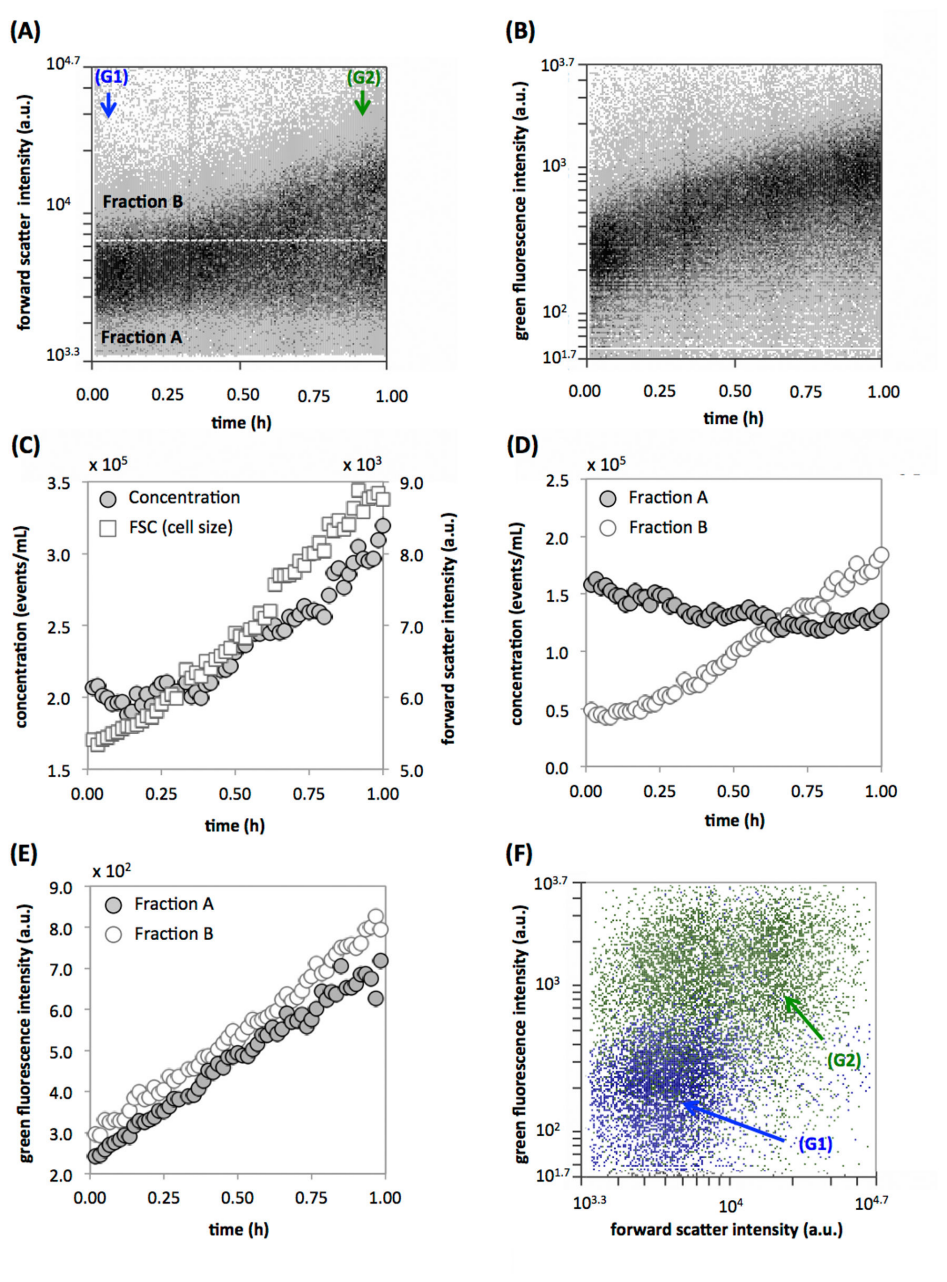
Initial growth response of late stationary phase *E. coli* cells inoculated into fresh growth medium. (**A**) Raw RT-FCM data of FSC intensity over time, with marked regions (G1) and (G2) showing data used for panels F. A set FSC value (white dotted line, indicating the line below which 80 % of particles lie at the beginning of the measurement) separates a fraction of small (Fraction A) and large (Fraction B) cells which are used for calculations in panels D and E. (**B**) Raw data of bacterial GFP fluorescence (ex. 488 nm/em. 530 nm) resulting from the enzymatic cleavage of CFDA. (**C**) Processed data (1-minute intervals) showing the changes in total cell concentration as well as median cell size (measured with forward scatter). (**D**) Changes in the cell concentrations of small (Fraction A) and large (Fraction B) cells (from panel A). (**E**) Changes in the green fluorescence intensity of small (Fraction A) and large (Fraction B) cells (from panel A). (**F**) FCM overlay density plot of the culture at the start (blue data; (G1)) and end (green data; (G2)) of the experiment.

As a third example, we show an experiment where bacterial cell size, measured as fluorescence-independent FSC, was used as our main dynamic readout, and enzymatic conversion of a specific fluorogenic substrate was a marker for cellular activity. The lag phase of bacterial batch cultures was recently described as the least understood bacterial growth phase, primarily due to the lack of adequate measuring techniques [25]. However, it is well known that dynamic changes occur in cell size during this period, and that flow cytometric scatter parameters are particularly sensitive to detect and quantify such change [25-27]. Figure 3A and 3B depict the raw data for the response of stationary phase *E. coli* cells that have been kept in batch culture for 6 days during the first 60 minutes after inoculation into fresh, pre-warmed growth medium, supplemented with carboxyfluorescein diacetate (CFDA). CFDA is converted to a fluorescent compound by the esterase enzyme and serves as a readout for cellular enzymatic activity [23]. The change in cell concentration over time revealed a period of approximately 0.25 - 0.3 h in which cell numbers stay roughly constant. After this phase, the whole population multiplied at a mean growth rate of 0.67 h^-1^ (Figure 3C). However, a continuous increase in the median FSC value prior to division clearly illustrates the onset of cell elongation within minutes after inoculation (Figure 3C), at least in a fraction of the population, corroborating similar results based on transcriptomics and studying nutrient assimilation [25]. As clearly visible in Figure 3A, different subpopulations in the culture respond differently to the newly available nutrients. To understand and describe this better, we applied a fixed FSC gate that included 80% of the culture at time 0 (“Fraction A” in Figure 3A) and tracked the development of this fraction relative to the events exceeding this FSC value (Fraction B) over time. Figure 3D shows the concentration dynamics of bacteria in each of these two FSC fractions. Evidently, the concentration of bacteria in the lower size class (Fraction A) decreased by about 3 x 10^4^ cells/mL during the first 0.5 h with a concomitant increase in larger sized particles (Fraction B), clearly indicating cell size increase in a part of the population. Thereafter, the cell concentration in Fraction A remains approximately constant at about 1.3 x 10^5^ cells/mL, suggesting that about 64% of the cells in the starting culture were unresponsive during the experimental period. This was not surprising, given the late stationary phase nature of the starting culture, where some dead or dormant cells should be expected. In contrast, nearly all cells demonstrated esterase substrate uptake and conversion (Figure 3B). When the fluorescence intensity of two FSC fractions was separately assessed, both fractions displayed similar CFDA conversion kinetics (Figure 3E). While this result suggests the presence of esterase activity in many cells, it does not necessarily indicate viability, since enzymes can still be functional when cells are dead [23]. This result is summarized in Figure 3F, showing a shift from a non-fluorescent cluster of small cells at the beginning (G1) to an overall fluorescent population in two FSC clusters at the end (G2). This example demonstrates how RT-FCM can be used to reveal and quantify a broad range of dynamic events in seemingly static samples and at low cell densities. 

### Algal treatment with hypochlorite

**Figure 4:**
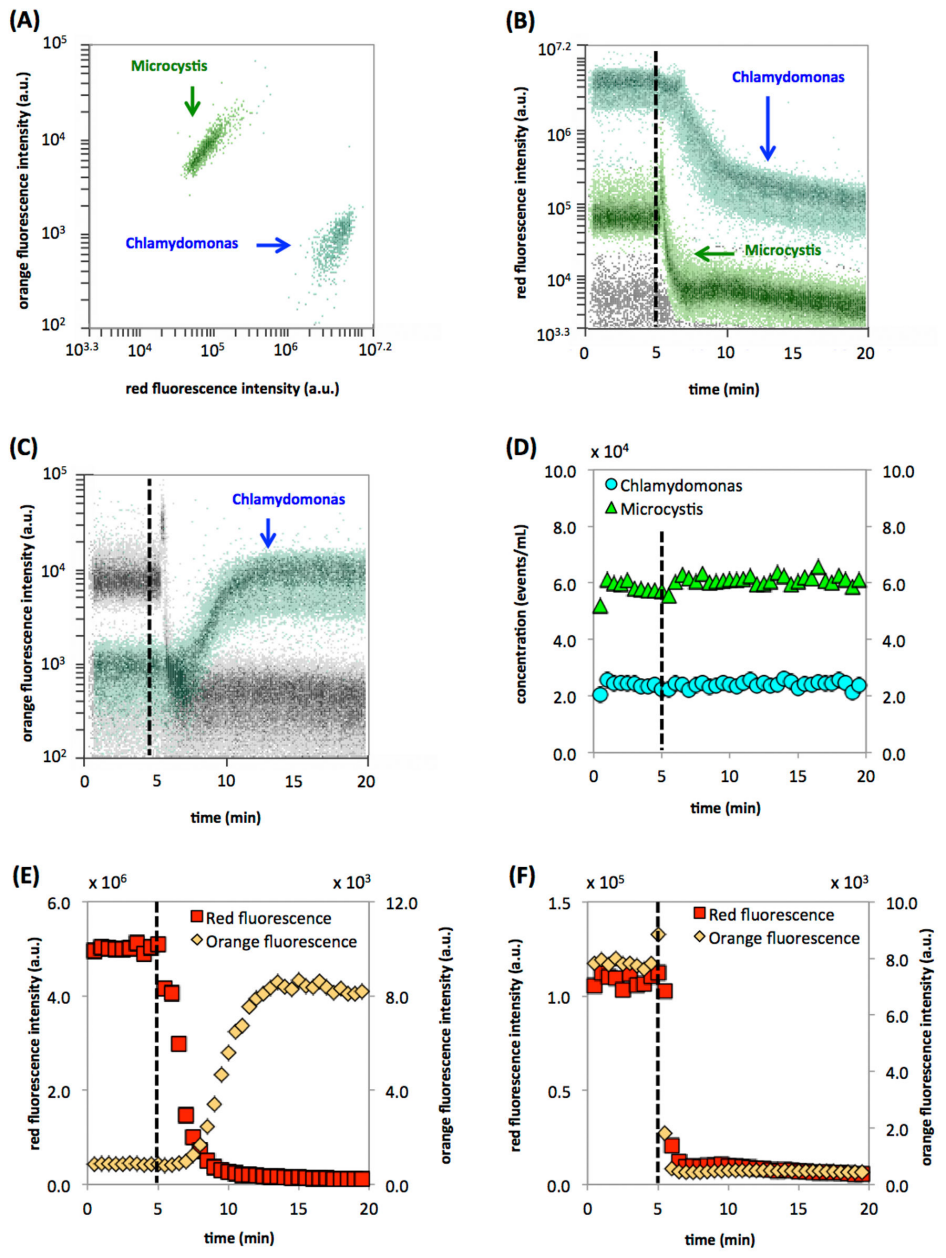
Treatment of a mixture of *Chlamydomonas reinhardtii* and *Mycrocystis aeruginosa* with hypochlorite during 20 minutes. (**A**) FCM density plot distinguishing the two populations in the starting mixture based on orange (ex. 488 nm/em. 585 nm) and red auto-fluorescence (ex. 488 nm/em. 670 nm). (**B**) Raw RT-FCM data of red auto-fluorescence intensity, resulting from the excitation of chlorophyll. Hypochlorite was added after 5 minutes (dotted line) (**C**) Raw RT-FCM data of orange auto-fluorescence intensity. (**D**) Processed data (30-second intervals) showing stable cell concentrations for both clusters during the experiment. (**E**) Changes in the red and orange fluorescence intensities of *C. reinhardtii*. (**F**) Changes in the red and orange fluorescence intensities of *M. aeruginosa*.

In the fourth example, we show an application that exploits the auto-fluorescence of chlorophyll in photosynthetic microorganisms, and the fact that this auto-fluorescence is sensitive to oxidative damage [28]. Our experimental culture was a mixture of algae (*Chlamydomonas reinhardtii*) and cyanobacteria (*Microcystis aeruginosa PCC 7806*) (Figure 4). This additionally allowed a demonstration of how differential responses in sub-clusters can be traced within a single experiment. Figure 4A shows the two organisms in their original state, with the *Chlamydomonas* notably high in red fluorescence from chlorophyll, and the cyanobacteria high in orange fluorescence from phycobillin pigments [29]. Figure 4B shows the raw red fluorescence data for both organisms, which decreased drastically in intensity after the addition of 0.6 g/L hypochlorite. Figure 4C shows the raw data for orange fluorescence, which displays a dissimilar response; the cyanobacterial orange fluorescence decreases, while the algal orange fluorescence increases noticeably. Although this damage was likely lethal to the microorganisms, it did not lead to disintegration of cells. Figure 4D shows that the cell concentration of both organisms remains stable throughout the experimental period. Figure 4E and Figure 4F shows the quantified red and orange fluorescence data for both microorganisms. It is known that damage to chlorophyll results in a decrease in red fluorescence [29]. This was clearly characterized for both *Chlamydomonas* (Figure 4E) and *Microcystis* (Figure 4F), where the high-resolution RT-FCM data allowed detailed tracking of these dynamic processes. Interestingly, the *Chlamydomonas* showed a clear increase in orange fluorescence (Figures 4C and 4E) following the decrease in red fluorescence. We opine that the increase in orange fluorescence is a direct result of oxidative damage to the algal photo-pigments, causing a shift in the fluorescence emission spectrum. However, conclusive evidence for this is lacking and further research would be required to understand this phenomenon. In the case of *Microcystis* (Figure 4F), this effect was not observed, with fluorescence increasing briefly directly upon chlorine addition, and both red and orange fluorescence decreasing rapidly thereafter. Moreover, Figures 4E and 4F clearly show the dissimilar response of the two clusters, suggesting that the *Chlamydomonas* cells were damaged at a slower rate than the *Microcystis* cells. It is evident that both the time of the initial response, as well as the kinetics of the fluorescence decrease, can only be detected with the high resolution obtained with an RT-FCM approach. 

## Discussion

RT-FCM is an approach that exploits the time-resolved nature of flow cytometric measurements, providing an FCM readout of a given experiment directly and in real-time, with an output of continuous data describing the processes occurring in the sample on single-cell level. RT-FCM has been used previously, and has been applied to both biotic and abiotic experimental systems [1,4-10]. However, to date the RT-FCM approach is virtually unused in microbiological applications. 

The above examples show applications of RT-FCM for four fundamentally different experimental questions, encompassing different microorganisms and different processes. We used pure cultures of *E. coli*, controlled mixtures of algae and cyanobacteria, and environmental samples as study objects; our primary readouts were (i) the fluorescence of a genetically encoded fluorescent protein that is produced upon stress, (ii) viability stains to visualize cell membrane damage, (iii) FSC to measure cell elongation and growth, and (iv) loss of cellular auto-fluorescence indicative of cell damage and death. All these measurements were performed using comparatively cheap and easy-to-use bench-top equipment that is nowadays widely available. 

The four examples above only touch upon the multi-disciplinary potential of the RT-FCM approach. Conventional flow cytometry allows considerable flexibility with respect to the analyzed material, from virus particles (ca. 0.1 μm) to mammalian cells (ca. 20 μm), to a range of inorganic particles (e.g., micro-beads), as well as the combination with a broad variety of fluorescent labels (e.g., fluorescent proteins, antibodies, stains and photo-pigments) [2,3,23]. The RT-FCM approach can be used for nearly all of these applications. For the examples shown in the present study, we utilized primarily intrinsic cytometric parameters such as intracellular fluorescence and auto-fluorescence, particle numbers, and scatter properties. Using intrinsic parameters is the most straightforward application of the technology, and, as demonstrated, these can yield valuable insights into selected processes. In addition to applications with intrinsic parameters, we present two poof-of-principle experiments showing that extrinsic staining reagents can also be used for studying dynamic processes with RT-FCM (Figure 2 and 3). However, the combination of staining reagents with RT-FCM experiments can raise several problems, such as adverse reactions of stain with the sample (e.g. DNA binding dyes inhibiting growth experiments), or experimental conditions that interfere with the staining process (e.g. chlorination leads to oxidation of dyes). Also, the reaction time of dyes has to be considered: it is evident that dyes that react in minute resolution will incorrectly report events that occur in second resolution during real-time analysis. On the one hand, many of these issues can be circumvented by the use of an intermediate staining robot [30,31] to separate the sample from the staining and measuring event. On the other hand, RT-FCM actually offers the perfect method to assess the time-dependent reaction of stains with cells under defined conditions. Staining efficacy is highly dependent on a variety of external factors, and optimizing staining protocols in individual experiments is very time consuming [30,32]. In this regard, RT-FCM offers an excellent opportunity for the optimization of existing staining protocols and the testing of new dyes. 

A further point of importance is the advantage of continuous data over discretely collected and processed samples. For highly dynamic processes that take place at a millisecond to low double-digit second resolution, it is virtually impossible to achieve data that is comparable to RT-FCM data in terms of information content by means of manual sampling over time. This is aptly illustrated with the dramatic response of the cyanobacterial culture to hypochlorite treatment in Figure 4. However, one may argue that for many processes, including most examples shown in our study, manual sampling at multiple time points yields similar data as RT-FCM. While this is in theory correct for many experiments that span several hours (e.g. Figures 1 - 3), there are several advantages that can be gained from continuous data. Firstly, the use of an automated experimental system liberates the researcher for other tasks. For example, the data presented in the present study was essentially generated from four flow cytometric runs. However, achieving the exact same 1-minute data resolution as shown in Figures 1 - 4 with manual FCM measurements would require no less than 520 individual sampling and processing events. Moreover, all kinetic calculations (e.g. growth rates, gene expression rates) benefit from the availability of a sufficient number of data points. In fact, it is safe to argue that any researcher studying a dynamic process would prefer having access to the continuous data at any given point, as opposed to a discrete number of samples at pre-selected intervals. Continuous data therefore obviates the need to choose time points for sampling, which is not trivial for unknown processes and often requires multiple rounds of trial and error to achieve the desired resolution. 

The main purpose of this study was not to produce new insights into the biological processes shown. Our goal was to provide examples that demonstrate the power and broad applicability of RT-FCM, and increase, or create, awareness and knowledge about it in fields where we see potential applications, especially microbiology. From a technological perspective, the RT-FCM method is straightforward, allowing its simple implementation for various applications and easy access for researchers from multiple fields. From a conceptual perspective, the time-resolved approach discussed here can greatly increase the usability of flow cytometry for new applications and fields.

## Methods

### RT-FCM configuration

The basic RT-FCM methodology is as follows: the sample injection port of a commercially available flow cytometer, that uses peristaltic pumping to achieve flow, is submerged directly in any experimental reactor of choice (see below for details). Time-resolved information of every FCM parameter is subsequently collected by continuously extracting sample from the reactor and feeding the suspension to the flow cytometer, using the FCM fluidics hardware. This allows the detection of dynamic processes and sudden events occurring in the reactor with millisecond resolution during experimental periods ranging from several minutes to multiple hours. The FCM software automatically records all variables in time, and information on any variable can easily be extracted for any given time point or period.

### Flow cytometry set-up

RT-FCM analysis was done with a standard bench-top Accuri C6 flow cytometer (Becton Dickinson Biosciences, Erembodegem, Belgium), which was calibrated according to the manufacturer’s recommendations. The FCM is equipped with a 488 nm laser with detectors for green fluorescence (533±30 nm), orange fluorescence (585±40 nm), red fluorescence (610±30 nm) and deep red fluorescence (<670 nm), as well as for forward scattered (FSC) and sideward scattered (SSC) light. The instrument has an additional 640 nm laser with a detector at 675±25 nm. The instrument’s sampling port was submerged directly in the experimental reactor, which differed for the various experiments (discussed below). Data were collected continuously using the instrument’s “unlimited run” function, with the sampling speed programmed on 16 - 66 µL/min at data acquisition rates varying between 50 - 300 events/second in all channels (see individual experiments). Trigger parameters and settings varied for different experiments and are therefore defined in the specific experimental sections below.

### Fluorescent reporter gene expression

Induction of the SOS response in *E. coli* K12 MG1655 was studied with a strain carrying a transcriptional fusion of the promoter of the *recA* gene to gfpmut2 on a plasmid [33]. A single colony was picked from a plate, and grown overnight in Luria Bertani (LB) medium (Sigma Life Science) amended with Kanamycin (50 µg/ml, Sigma). From this culture, a 100-fold diluted sub-culture was prepared in the same medium and grown to exponential phase. This culture was then diluted in 10 ml sterile LB medium (10%) in a 15 ml culture tube (Sarstedt) to achieve a final cell concentration of 1.25 x 10^5^ cells/ml. During the experiment, the culture was aerated using a magnetic stirrer, and temperature control was achieved by immersion in a water bath at 37 °C. The culture tube was connected directly to the FCM via the instrument’s sampling port. After approximately one minute of measurements, 4 µg/ml ciprofloxacin (Fluka) was added to the culture, which has been reported to correspond to 50% of the minimum inhibitory concentration of the organism [34]. The instrument trigger was set on FSC and gates were used to distinguish bacteria from background and resolve the bacterial GFP fluorescence (533±30 nm) and FSC over time. Events were collected continuously during two hours at a flow rate of 16 µL/min and an average event rate of 128 events/second, and were subsequently gated throughout the entire period using adjacent gates with 1-minute gate widths (Figure S1). Growth rates were calculated from the linear portions of the graph depicting the natural logarithm of cell concentration changes over time (Figure 1C) with the equation µ = Δln(cells/mL)/Δt, as described previously (Berney et al., 2006). Rates of change in fluorescence and scatter were calculated using the slope of 7 adjacent data points (7 minutes) with 1-minute progression.

### Fluorescent labeling of heat-induced cell damage

Heat-induced damage to bacterial membranes was illustrated by exposing an autochthonous microbial community in a river water sample (Chriesbach, Dübendorf) to continuously increasing temperatures. A 5 mL water sample was pre-warmed (35 °C, 10 minutes) and then stained with a 10 µL/mL of a stock solution of SYBR Green I (SG; Invitrogen) and propidium iodide (PI; Invitrogen), comprising 100x diluted SG (from original stock solution of 10’000x concentrate) and 300 µM PI in TRIS Buffer [30]. The stained sample was incubated (35 °C, 10 minutes) prior to the start of the RT-FCM measurements to allow complete staining of all bacterial cells. During the experiment, the stained sample was continuously mixed using a magnetic stirrer, and temperature control was achieved by immersion in a water bath with an adjustable temperature regulator. The sample vial was connected directly to the FCM via the instrument’s sampling port. After 5 minutes of data collection at 35 °C, the temperature was increased from 35 °C to 70 °C during 60 minutes at a gradual rate of approximately 0.5 - 1 °C per minute, with continuous data collection during this period. The instrument trigger was set on FL1 (533±30 nm) and gates were used to distinguish intact bacteria from background (Figure 2A; see also 21) as well as to distinguish between low (LNA) and high (HNA) nucleic acid content bacteria [32], and to resolve the changes in bacterial fluorescence over time. Events were collected continuously during 60 minutes at a flow rate of 16 µL/min and an average event rate of 256 events/second and subsequently gated throughout the entire experimental period with adjacent gates with 1-minute intervals. 

### Bacterial lag phase response

Analysis of the initial growth response of a stationary bacterial culture was studied using *E. coli* K12 MG1655 inoculated in fresh growth media. A late stationary phase culture was prepared by cultivating *E. coli* in 30 % LB medium at 37 °C for 3 days with shaking at 220 rpm and subsequently leaving this culture stationary at room temperature for another 3 days. For the experiment, the stationary culture was diluted 10,000-fold (final concentration = 2 x 10^5^ cells/mL) in 37 °C pre-warmed fresh LB medium (30 %) amended with carboxyfluorescein diacetate (CFDA) as prescribed by the manufacturer. The culture was constantly aerated by magnetic stirring and a RT-FCM measurement was initiated within 1 minute after inoculation. The instrument trigger was set on FSC and gates were used to distinguish bacterial cells from background and to resolve green fluorescence intensity resulting from enzymatic conversion of CFDA. Data were collected continuously during 60 minutes at a flow rate of 16 µL/min and an average event rate of 70 events/second and subsequently gated throughout the entire experimental period with adjacent gates with 1-minute intervals. 

### Algal disinfection with hypochlorite

A pure culture of the cyanobacteria *Microcystis aeruginosa PCC 7806* (6.0 x 10^4^ cells/mL final concentration) was mixed with the algae *Chlamydomonas reinhardtii* (2.5 x 10^4^ cells/mL final concentration). Preliminary FCM analysis of the mixture revealed two clearly distinguishable clusters based on the fluorescence intensity of their respective photo-pigments (Figure 4A). For the experiment, a 5 mL sample of this mixture was continuously stirred at room temperature and a RT-FCM measurement was initiated as described above. After 5 minutes of data collection, the sample was treated with 0.6 g/L sodium hypochlorite (Sigma). The instrument trigger was set on FSC, while red fluorescence (FL3; 670 nm) as well as orange fluorescence (FL2; 585 nm) were used as the main dynamic readouts. Data were collected continuously during 20 minutes at a flow rate of 33 µL/min and an average event rate of 90 events/second and subsequently gated throughout the entire experimental period with adjacent gates with 0.5-minute intervals. For data analysis, the two distinct clusters representing the two organisms were separately gated on a density plot (FL3 vs time). Thereafter, the time-resolved data of cell concentration and red (FL3; 670 nm) and orange (FL2; 590 nm) fluorescence intensity for each cluster were separately collected.

### FCM gating strategy and data management

All of the RT-FCM data sets from the various experiments were gated with a similar approach, outlined in Figure S1. For each experiment a trigger parameter and threshold value was used for data collection, depending on the characteristics of the specific samples as described above. Subsequently, data were collected and resolved against time for all FCM parameters. If required, an additional selective gate was used to separate the main sample signals from unwanted background signals originating from instrument noise or the media in which the samples were suspended. In some instances, specific sub-populations were additionally selected (e.g., intact cells and LNA/HNA fractions (Figure 2) or the algae and cyanobacteria clusters (Figure 4)). The parameters of interest were then processed by gating on a density plot of a selected parameter vs time using adjacent gates, having gate widths of either 0.5-minutes (Figure 4) or 1-minute (Figure 1 - 3). From each of these gates, quantitative data (particle concentration and median parameter values) was extracted and further processed with MS Excel. The original FCM files for all the experiments were deposited at Flowrepository.org. 

## Supporting Information

Figure S1 **Example of the RT-FCM gating strategy.** For details, see the description above. Note that the FCM density plots are original data from the experiment shown in Figure 2 (manuscript), but the gates are not the actual gates used for data analysis. The gates were manually inserted afterwards with illustration software to make the gating strategy clear.
(DOCX)
