# Supplementary material for: Monitoring of Dynamic Microbiological Processes Using Real-Time Flow Cytometry
Source: PLoS One. 2013 Nov 14;8(11):e80117. doi: 10.1371/journal.pone.0080117 (PMC3828236; doi:10.1371/journal.pone.0080117)
Supplement: Figure S1 — Example of the RT-FCM gating strategy. For details, see the description above. Note that the FCM density plots are original data from the experiment shown in Figure 2 (manuscript), but the gates are not the actual gates used for data analysis. The gates were manually inserted afterwards with illustration software to make the gating strategy clear. (DOCX) [file pone.0080117.s001.docx]

Supplementary information for:

**Monitoring of dynamic microbiological processes using real-time flow cytometry**

Markus Arnoldini, Tobias Heck, Alfonso Blanco-Fernández & Frederik Hammes

**RT-FCM gating strategy**

**Example: Heat induced membrane damage (Figure 2 in manuscript)**

1. A main (trigger) parameter for the experiment is selected and a threshold value is applied to this parameter to enable detection of the sample. In this case, the parameter was green fluorescence (FL1) and a threshold value was applied based on experience with this specific type of sample. **Figure S1A** shows a density plot of the sample at the beginning of the experiment, with blank space on the left indicating the threshold value.
2. Additional gates are prepared, based on the requirements of the specific experiment. In this example, **Figure S1B** show gates for all of the “intact” cells (P1) as well as the fraction of high nucleic acid (HNA) content bacteria (P2) and low nucleic acid (LNA) content bacteria (P3). The researcher, based on experience and controls, draws these gates manually. For this specific example, see Prest et al. (2013), Berney et al. (2007) and Ramseier et al. (2011) and Hammes et al. (2012) for more information on the gating strategy.
3. The RT-FCM experiment is then performed for a selected time period, and the main parameter is plotted in a density plot against time. **Figure S1C** shows the time period of 1 hour and green fluorescence intensity (FL1). Note that this is un-gated raw data, that the RT-FCM data is not restricted to the main parameter (FL1), but can be applied to all FCM parameters.
4. After the experiment, selected gates are applied to the raw data set to assess specific clusters or groups of interest. **Figure S1D** shows the RT-FCM data for all “intact cells” (from gate P1), **Figure S1E** shows the data for “intact LNA cells” (from gate P3), and **Figure S1F** shows the data for “intact HNA cells” (from gate P2). Again, this is not restricted to one parameter or limited gates.
5. The entire RT-FCM data set is then gated on the parameter vs time plot. In our experiments we selected for a resolution of 1-minute gate widths, illustrated in **Figures S1G-I**. Note that the gates in Figures S1G-I are not the actual gates used in our analysis. The frequency of the gates is the choice of the researcher, but higher frequency automatically allows for higher resolution in the data. From these gates, the required quantitative data are collected for further processing. In the present study, we utilized primarily the cell concentration within the gate as well as the median value for the parameter.
6. Notably, these steps are not limited to the specific software of the instrument that was used for the analysis. The experimental data can be exported as a CSV or FCS file and can then be processed with any number of statistical software packages available for FCM data analysis.


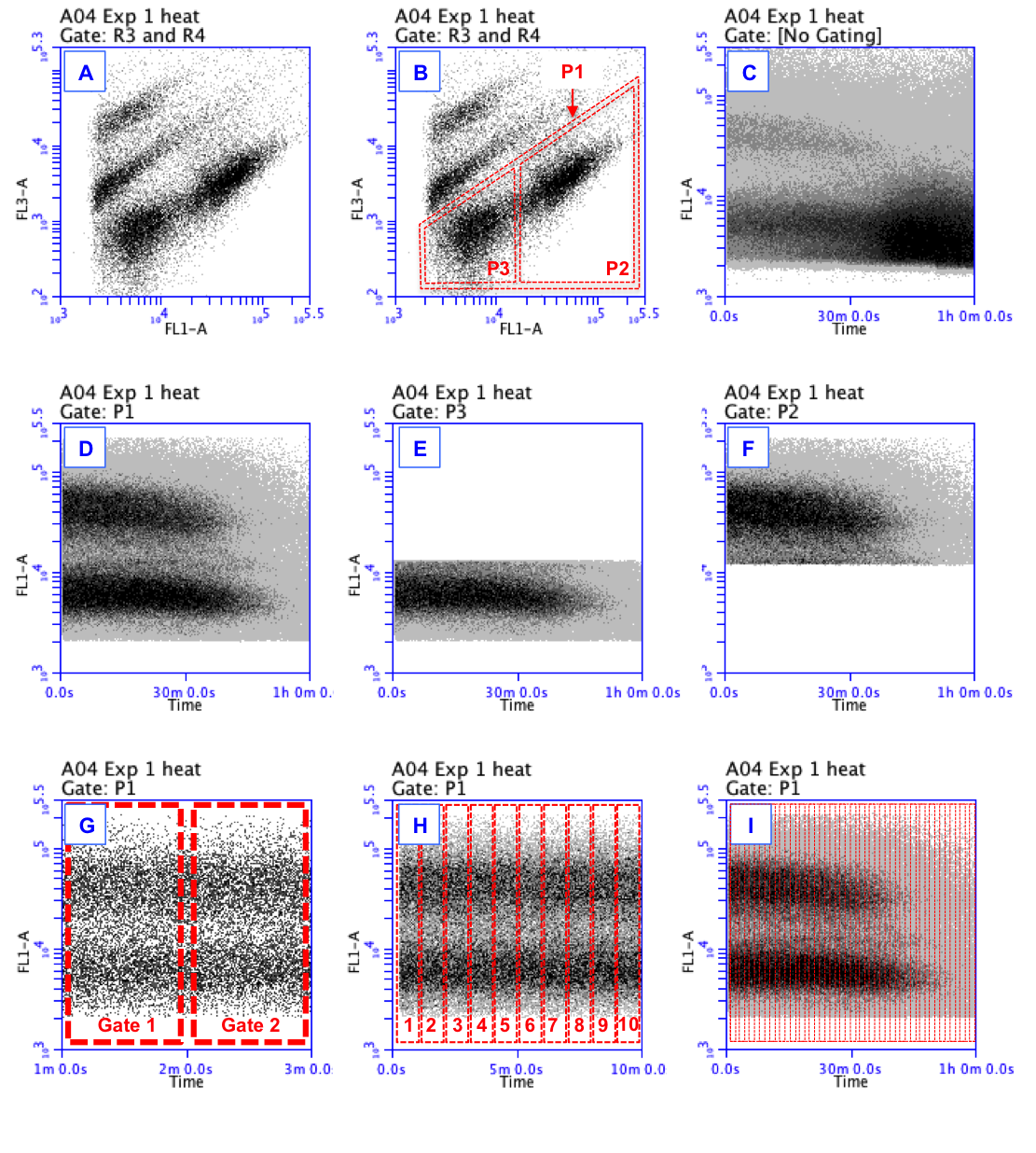


**Figure S1.** Example of the RT-FCM gating strategy. For details, see the description above. Note that the FCM density plots are original data from the experiment shown in Figure 2 (manuscript), but the gates are not the actual gates used for data analysis. The gates were manually inserted afterwards with illustration software to make the gating strategy clear.

**References**

1. **Berney, M. *et al.*** (2007) Assessment and interpretation of bacterial viability by using the LIVE/DEAD BacLight kit in combination with flow cytometry. *Applied and Environmental Microbiology* 73 (10), 3283-3290.
2. **Hammes, F. *et al.*** (2012) Development and laboratory‐scale testing of a fully automated online flow cytometer for drinking water analysis. *Cytometry Part A* (2012).
3. **Prest, E. *et al.*** (2013) Monitoring microbiological changes in drinking water systems using a fast and reproducible flow cytometric method. *Water Research* (in press).
4. **Ramseier, M.K., *et al.*** (2011) Kinetics of membrane damage to high (HNA) and low (LNA) nucleic acid bacterial clusters in drinking water by ozone, chlorine, chlorine dioxide, monochloramine, ferrate(VI), and permanganate. *Water Research* 45 (3), 1490-1500.
